# Cascade Reaction in Human Live Tissue Allows Clinically Applicable Diagnosis of Breast Cancer Morphology

**DOI:** 10.1002/advs.201801479

**Published:** 2018-11-27

**Authors:** Tomonori Tanei, Ambara R. Pradipta, Koji Morimoto, Motoko Fujii, Mayumi Arata, Akihiro Ito, Minoru Yoshida, Elena Saigitbatalova, Almira Kurbangalieva, Jun‐ichiro Ikeda, Eiichi Morii, Shinzaburo Noguchi, Katsunori Tanaka

**Affiliations:** ^1^ Department of Breast and Endocrine Surgery Graduate School of Medicine Osaka University 2‐2‐E10 Yamadaoka Suita Osaka 565‐0871 Japan; ^2^ Biofunctional Synthetic Chemistry Laboratory RIKEN Cluster for Pioneering Research 2‐1 Hirosawa Wako Saitama 351‐0198 Japan; ^3^ Osaka Women's Junior College 3‐8‐1 Kasugaoka Fujiidera Osaka 583‐8558 Japan; ^4^ Seed Compounds Exploratory Unit for Drug Discovery Platform RIKEN Center for Sustainable Resource Science 2‐1 Hirosawa Wako Saitama 351‐0198 Japan; ^5^ Chemical Genomics Research Group RIKEN Center for Sustainable Resource Science 2‐1 Hirosawa Wako Saitama 351‐0198 Japan; ^6^ School of Life Sciences Tokyo University of Pharmacy and Life Sciences 1432‐1 Horinouchi Hachioji Tokyo 192‐0392 Japan; ^7^ Biofunctional Chemistry Laboratory A. Butlerov Institute of Chemistry Kazan Federal University 18 Kremlyovskaya Street Kazan 420008 Russian Federation; ^8^ Department of Pathology (C3) Graduate School of Medicine Osaka University 2‐2 Yamadaoka Suita Osaka 565‐0871 Japan; ^9^ GlycoTargeting Research Laboratory RIKEN Baton Zone Program 2‐1 Hirosawa Wako Saitama 351‐0198 Japan

**Keywords:** acrolein, breast cancer, breast‐conserving surgery, imaging, TAMRA phenyl azide

## Abstract

Clean operating margins in breast cancer surgery are important for preventing recurrence. However, the current methods for determining margins such as intraoperative frozen section analysis or imprint cytology are not satisfactory since they are time‐consuming and cause a burden on the patient and on hospitals with a limited accuracy. A “click‐to‐sense” probe is developed based on the detection of acrolein, which is a substance released by oxidatively stressed cancer cells and can be visualized under fluorescence microscopy. Using live breast tissues resected from breast cancer patients, it is demonstrated that this method can quickly, selectively, and sensitively differentiate cancer lesion from normal breast gland or benign proliferative lesions. Since acrolein is accumulated in all types of cancers, this method could be used to quickly assess the surgical margins in other types of cancer.

Breast cancer is a disease with a high prevalence, affecting many women worldwide at some point during their lives.^1^ Currently, breast‐conserving surgery (BCS) is a good option for women with early‐stage breast cancer and can be combined with postoperative radiation therapy to reduce the risk of ipsilateral breast tumor recurrence (IBTR). A meta‐analysis performed by the Early Breast Cancer Trialists' Collaborative Group (EBCTCG) showed that postoperative radiation therapy significantly reduces IBTR and 15‐year breast cancer mortality following BCS.^2^ However, surgical margins are important as well. In 2014, the Society of Surgical Oncology and American Society for Radiation Oncology published a “consensus guideline” stressing the importance of management margins for BCS in Stage I and II patients. This guideline is based on a meta‐analysis of 28162 patients from 33 studies that found that positive margins, defined on invasive ductal carcinoma (IDC) or ductal carcinoma in situ (DCIS), which were analyzed by postoperative pathologic methods, are associated with at least a two‐fold increase in IBTR. Thus, negative margins are associated with low rates of IBTR and have the potential to decrease re‐excision rates.^3^ However, there is as of yet no established global standard for real‐time intraoperative margin management in BCS.

Intraoperative pathologic methods including frozen section analysis and imprint cytology have the potential to lower rates of positive margins.^4^ But these methods cannot be generally applied to current BCS in hospitals worldwide, due to the increase of operating time resulting from the pathological procedure and the increased workload for pathologists while making frozen sections (**Figure** **1**A).

**Figure 1:**
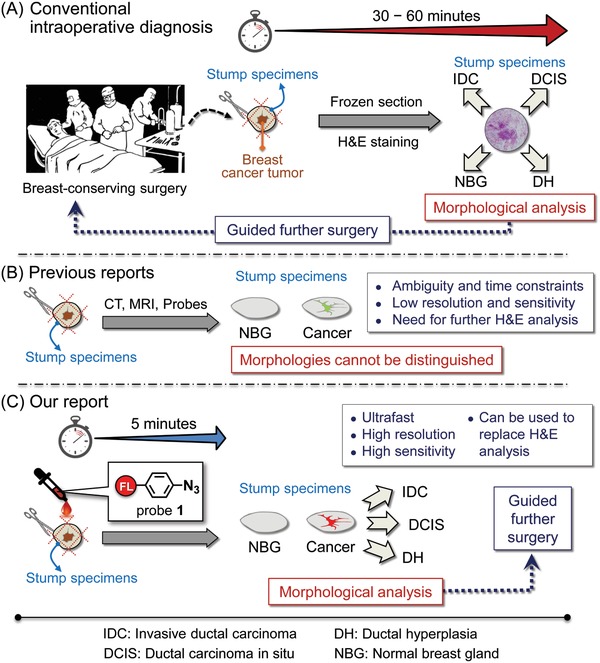
Schematic presentation of intraoperative diagnosis. A) The pathology‐based method currently used in BCS. Frozen samples of breast cancer stumps are analyzed using microscopy with H&E staining. Further surgery is guided based on morphological analysis, i.e., depending on the IDC, DCIS, DH, and NBG regions. Several pathological procedures require about 1 h. B) Recently emerging techniques use CT, MRI, and chemical probes. The methods can image live cancer tissues, but still have problems associated with sensitivity and selectivity. Importantly, these methods cannot image the morphology of various cancer stages, and still require pathology to confirm their diagnosis (hence they are unlikely to replace the conventional H&E method as described in (A)). C) The “click‐to‐sense” method described in this paper allows the diagnosis of cancer morphology with high sensitivity and selectivity by simply treating the live tissues with chemical probe **1** for 5 min and then by fluorescent microscopy. Our new method could substitute for conventional pathological diagnosis.

Under such circumstances, new methods or techniques have been sought, and several have recently emerged for real‐time intraoperative management of breast margins using live tissues. These methods include MarginProbe, which senses electromagnetic waves reflected from breast cancer regions,^5^ microcomputed tomography (CT),^6^ and ex vivo magnetic resonance imaging (MRI)^7^ (Figure 1B). These methods involve imaging the macrosize of cancer lesions, but clear‐cut cancer morphology and localization even at the cell level in live tissues cannot be evaluated. In fact, the false‐positive rate using the MarginProbe device was higher (53.6%) than that in the control arm (16.6%) in a randomized prospective study.^5^ A chemistry‐based fluorescent probe that is selectively activated in the presence of γ‐glutamyl transferase, which is overexpressed in cancer lesions, has also been reported (Figure 1B).^8^ This method relies on the time‐dependent increase of fluorescence, so sample‐to‐sample reproducibility (i.e., cancer selectivity due to high fluorescence backgrounds and fluorescent spreading) has made breast cancer surgeons hesitant to use it in real BCS. More critically, this chemical method cannot diagnose cancer morphology. It is sometimes assumed that these methods will be useful for actual breast conserving surgery, but since they cannot be used to determine cancer morphology in live tissues, they are unlikely to replace the pathologic methods using frozen tissues. In fact, none of the newly emerging methods has been successfully applied to real‐time intraoperative margin management in BCS. Thus, there is still a need for new methods to allow the rapid, selective and sensitive diagnosis of various cancer morphology using live tissues.

Acrolein, a highly toxic α,β‐unsaturated aldehyde,^9^ has long been known as a biomarker associated with a range of disorders related to oxidative stresses, including cancers. It is produced through the enzymatic oxidation of threonine or polyamines^10^ and is also generated during reactive oxygen species (ROS)‐mediated oxidation of highly unsaturated lipids.^11^ It is sometimes generated on a few 100 × 10^−6^ m scale in oxidatively stressed cells^12^ and is more toxic to cells than ROS such as hydrogen peroxide (H_2_O_2_) or hydroxyl radicals (•OH), the major oxidative stress factors associated with a variety of disorders.^13^ In fact, mass spectrum analysis of the liquid‐phase extracts of cell lysates has shown that cancer cells produce acrolein.^14^

We recently developed a “click‐to‐sense” probe **1** for detecting acrolein, based on an acrolein/azide “click” reaction (Figure 1C).^15^ In contrast to previous methods for detecting acrolein, such as derivatization/HPLC,^16^ mass spectra analysis,^14^ or mAb detection of the acrolein‐lysine adducts *N*‐(3‐formyl‐3,4‐dehydropiperidino)lysine (FDP‐lysine),^17^ our method can sensitively detect the presence of acrolein using fluorescence, so that even acrolein present at the nm level can be detected directly within live cells. Thus, we found that the azide functionality in probe **1** participated smoothly in the previously unexplored 1,3‐dipolar cycloaddition reaction with acrolein generated from the cells, producing 1,2,3‐triazoline derivatives (**Figure** **2**A). The 1,2,3‐triazoline derivatives then decomposed into the corresponding diazo compounds in or on the surface of the cells, and that these compounds immediately and nondiscriminately conjugated with the cell constituents to anchor fluorescence within the cells. The new method is noteworthy for its simplicity: oxidatively stressed cells are treated with probe **1**, and the acrolein/probe **1** “clicked” conjugates anchored within the cells can be directly measured by fluorescence readout. The intensity of the fluorescence is proportional to the acrolein concentration generated by the cells.

**Figure 2:**
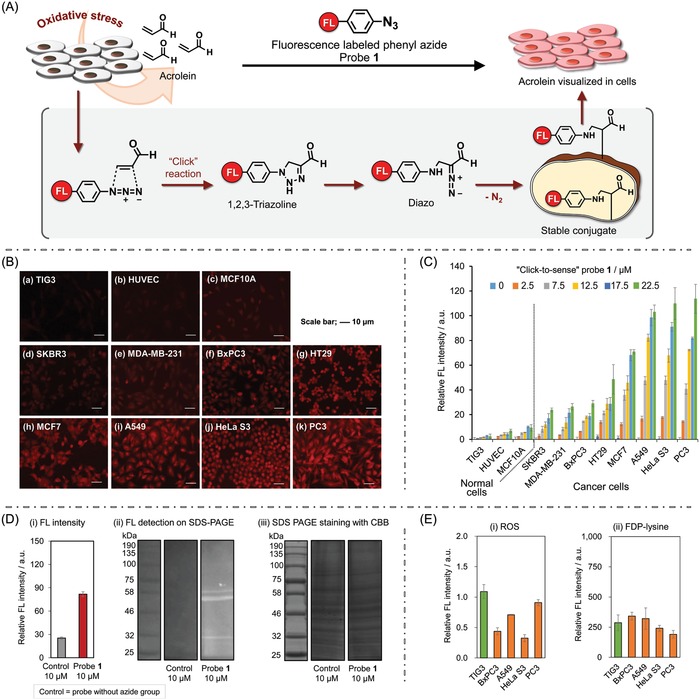
A) “Click‐to‐sense” the cancer: method and mechanism. Fluorescently labeled azide **1** smoothly reacts with acrolein generated by cancer cells through a 1,3‐dipolar cycloaddition reaction (acrolein/azide “click” reaction). 1,2,3‐triazole as the “clicked” product decomposes into diazo compounds, which react with cell constituents to anchor the fluorescence label within the cells. The acrolein concentration is analyzed in a simple way by fluorescence readout at the whole‐cell level. B) Discrimination of cancer cells from normal cells by “click‐to‐sense” probe **1**. Eleven cell lines were treated with our probe at 37 °C for 30 min. The cells were fixed and fluorescence was recorded by SpectraMax M2e, Molecular Devices. Fluorescence images of cells after incubation with 22.5 × 10^−6^ m of probe **1** (see Figure S1, Supporting Information, for images after treatment with other concentrations of **1**). (a) TIG3; (b) HUVEC; (c) MCF10A; (d) SKBR3; (e) MDA‐MB‐231; (f) BxPC3; (g) HT29; (h) MCF7; (i) A549; (j) HeLa S3; and (k) PC3. The scale bar indicates 10 µm. C) Fluorescence intensity observed for each cell lines at the concentration range of 0–22.5 × 10^−6^ m of probe **1**. Left to right: TIG3, HUVEC, MCF10A, SKBR3, MDA‐MB‐231, BxPC3, HT29, MCF7, A549, HeLa S3, and PC3. Fluorescence intensity was normalized as that emitted by 10 000 cells. D‐i) Total fluorescence intensity of HeLa S3 cell lysates (see method in Supporting Information); (ii) the SDS–PAGE images by fluorescence detection of HeLa S3 cell lysates treated with 10 × 10^−6^ m of probe **1** and control probe without azide group (see also Figure S2, Supporting Information). Cell constituents are labeled by the probe **1** based on the mechanism described in (A); (iii) the SDS–PAGE by coomassie staining (see method in Supporting Information). E) (i) ROS and (ii) FDP‐lysine analysis of the lysates of the selected cell lines (left to right: TIG3, BxPC3, A549, HeLa S3, and PC3). These methods (see Supporting Information) cannot selectively detect cancer.

Considering that acrolein is produced in significant amounts in cancer from cells undergoing oxidative stress, we hypothesized that our “click‐to‐sense” probe **1** could allow us to selectively and sensitively image cancer cells in live tissues. We realized that if the selectivity could be ensured, the method could be used as a discriminative, inexpensive and easy‐to‐perform method for cancer sensing during surgery.

In this paper we report, using our method, the first ever success of “real‐time” intraoperative assessment using resection stumps from live tissues. Our probe overcomes many of the limitations of other techniques so far reported (Figure 1C). Unlike other methods, it can discriminate, in a clear‐cut manner, IDC and DCIS from normal breast gland (NBG) and ductal hyperplasia (DH), and sensitively visualize cancer morphology and localization (even at the cell level) in the resection stump during surgery. Our probe could be used alongside with current breast‐conserving surgery in hospitals worldwide, eliminating the need for laborious, expensive, and time‐consuming pathological procedures using frozen tissues.

*“Click‐to‐Sense” Probe **1** Clearly Images the Acrolein Generated from Cancer Cells In Vitro*: We began by investigating whether our “click‐to‐sense” probe **1** could selectively image cancer cells in vitro. In this study, eight cancer cell lines such as PC3 (human prostate cancer cells), HeLa S3 (human cervical cancer cells), A549 (adenocarcinomic human alveolar basal epithelial cells), MCF7 (human breast cancer cells), HT29 (human colon cancer cells), BxPC3 (human pancreatic cancer cells), MDA‐MB‐231 (human breast cancer cells) and SKBR3 (human breast cancer cells), as well as three normal cell lines such as MCF10A (normal human mammary cells), HUVEC (human umbilical vein endothelial cells), and TIG3 (normal human diploid cells) as control were used. Following established procedures,^15^ cells were treated with five concentrations of probe **1** (2.5 × 10^−6^, 7.5 × 10^−6^, 12.5 × 10^−6^, 17.5 × 10^−6^ and 22.5 × 10^−6^ m) at 37 °C for 30 min, and fluorescent intensity was recorded by spectrofluorometer (SpectraMax M2e; Molecular Devices). Fluorescence intensity was normalized for each cell line by the number of 10000 cells (Figure 2B,C and Figure S1, Supporting Information).

While the MCF10A, HUVEC, and TIG3 cells showed only negligible fluorescence at the concentration range of 0–22.5 × 10^−6^ m of our probe, a notable dose‐dependent increase of fluorescence was observed for all the cancer cells types. The highest fluorescence intensity was observed in PC3, HeLa S3, A549 and MCF7, followed by the HT29, BxPC3, MDA‐MB‐231 and SKBR3 cell line series (Figure 2C). Thus, different cancer cell lines exhibit different fluorescence intensities (implying different levels of increased acrolein production) upon treatment with our probe. Consistent with the proposed mechanism of how the “click‐to‐sense” probe **1** senses the acrolein generated from cancel cells (see Figure 2A), intracellular and membrane constituents (e.g., proteins or lipids) of the representative cancer cells were labeled by probe **1**, based on size‐partitioning filtration (Figure 2D‐i) and SDS‐PAGE analysis of the lysates of the probe **1**‐treated HeLa S3 cells (Figure 2D‐ii,iii, and Figure S2, Supporting Information).

It should be noted that these cancer cells could not be selectively imaged by either ROS probe (ROS‐ID Total ROS detection kit, Enzo) or conventional acrolein probe (detecting FDP‐lysine produced on cellular proteins)^17^, ^18^ (Figure 2E). Therefore, the data in Figure 2 show that our “click‐to‐sense” probe **1** can sensitively discriminate cancer from normal cells based on the in situ‐produced acrolein in or on live cells.

*“Click‐to‐Sense” Probe* ***1*** *and Hoechst staining for Differentiation between Live Breast Cancer, NBG, and DH Tissues*: To study the ability of the probe to be used in vivo, we performed analysis on 30 cancer (20 IDC and 10 DCIS) tissues, 30 NBG tissues, and 5 DH tissues from 30 breast cancer patients who underwent breast surgery during the period from March 2017 to March 2018 at Osaka University Hospital, Osaka, Japan, using a double fluorescence staining method with both our “click‐to‐sense” probe **1** and Hoechst 33 342 + 33 258. The live tissues were cut by Tissue Matrix Chamber to create a flat surface, dipped into a 5 × 10^−6^, 10 × 10^−6^, or 20 × 10^−6^ m solution of the “click‐to‐sense” probe **1** for 5 min, and rinsed with buffer (**Figure** **3**A). The resulting tissues were then directly analyzed by Keyence BZ‐X710 equipped with the optical sectioning algorithm system to obtain both gross pictures and double fluorescence staining images (Figure 3B). The Keyence sectioning algorithm allows user to obtain clear images without fluorescence blurring, in a way comparable to those captured on a laser confocal microscope, but in a fraction of the time and without the damaging effects of a laser. In optical sectioning the excitation light is passed through an electronic light modulator and is projected in a grid pattern onto only the focused areas of specimen. These focused sections are then combined to create a clear image that is free of out‐of‐focus fluorescence (Figure 3C). Since this does not require the use of any lasers of high intensity illumination, damage to the sample is minimized.^19^

**Figure 3:**
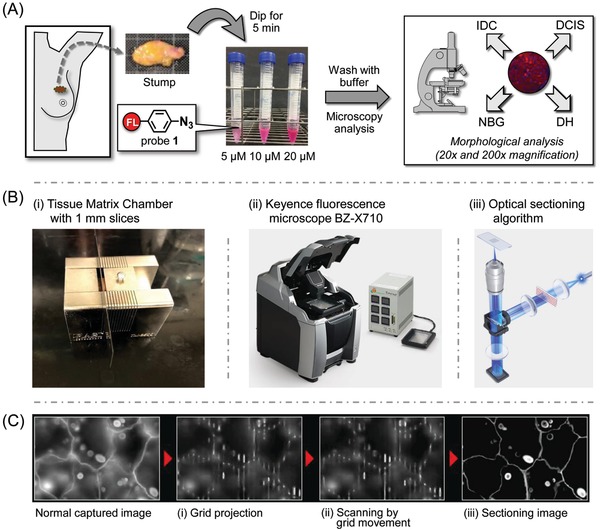
A) Schematic procedure for labeling live tissues (breast cancer stumps) with probe **1**. B) (i) A Tissue Matrix Chamber was used to slice fresh tissue; (ii) Keyence fluorescence microscope BZ‐X710; (iii) Optical sectioning algorithm system. C) The BZ‐X710 uses structured excitation light to scan the specimen, making it possible to capture clear images with no fluorescence blurring. (i) Excitation light from a metal halide lamp is passed through an electronic projection element, which projects the light onto the specimen in a grid pattern. The grid is only projected onto the focused areas of the specimen. (ii) Moving the grid causes the specimen to be scanned, allowing the capture of multiple images of the specimen. (iii) Extracting only the areas that the grid is projected onto from the multiple captured images prevents the effect of fluorescence blurring in the vertical direction. This produces clear images in which only the signal from the focused surface is extracted. Reproduced with permission. Copyright 2018, KEYENCE CORPORATION.

Representative images are shown in **Figure** **4**A‐i (IDC and NBG) and Figure 4A‐ii (DCIS and NBG). In both the gross pictures and fluorescent images, the breast cancer tissues (IDC or DCIS) were more highly stained by our probe than NBG tissues. The statistical significance of the tumor selectivity is shown in Figure 4B. The mean fluorescence intensity of breast cancers (IDC or DCIS, with their identification based on morphological analysis as discussed below) was significantly higher than that of NBG tissue in a statistically significant dose‐dependent manner at probe concentrations of 10 × 10^−6^ and 20 × 10^−6^ m. When we optimized the dividable threshold value of fluorescence intensity, the sensitivity and specificity were 90% and 97% at the probe concentration of 10 × 10^−6^ m; and were 97% and 97% at the probe concentration of 20 × 10^−6^ m, for binary classification between breast cancers and NBG, respectively (Figure 4B). This sensitivity and specificity have never been achieved by any other method. Importantly, our “click‐to‐sense” probe **1** labeled the breast cancer tissues with similar mean fluorescence intensity, regardless of IDC subtypes such as estrogen receptor (ER) or human epidermal growth factor receptor 2 (HER2) status, so as of DCIS subtypes for different ER status (Figure S4, Supporting Information).

**Figure 4:**
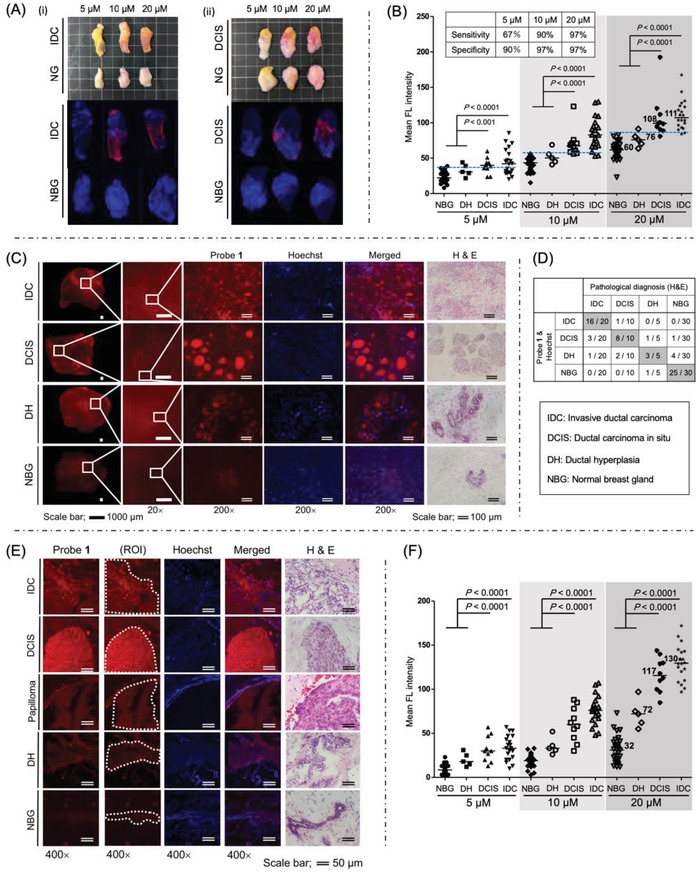
Microscopic images of live breast cancer (IDC, DCIS, DH) and normal breast gland (NBG) tissues labeled by “click‐to‐sense” probe **1** [for 5 min at 5 × 10^−6^, 10 × 10^−6^, and 20 × 10^−6^ m (red)] and Hoechst (blue). A) Pictures and double‐staining microscopic images of (i) IDC and NBG, (ii) DCIS and NBG. We defined ROI as shown in Figure S3 (Supporting Information). B) Statistical analysis of cancer sensitivity and selectivity among IDC, DCIS, DH, and NBG. Statistical test: *P*‐values, Mann‐Whitney U‐test; Bars, median. C) Expansion of images; Morphology of IDC, DCIS, DH, and NBG can clearly be detected by 20× and 200× (highly magnified fluorescent images) being labeled by probe **1** at 20 × 10^−6^ m. The detection of cancer morphology was consistent with H&E staining images of the frozen sections prepared from the same anonymized tissue samples. D) Analyzing 30 live cancer tissues (20 IDC and 10 DCIS), 30 adjacent live NBG tissues, and 5 adjacent live DH tissues from 30 breast cancer patients. Comparison of morphological analysis between “click‐to‐sense” method (by directly imaging the **1**‐labeled live tissues by microscopy (20×) and (200×)) with conventional H&E pathological method, performed by pathologist in an anonymized form. H&E analysis was performed by preparing the frozen sections from the same tissue samples. E) Confocal microscopic images (400×) of the frozen sections from the same samples prepared in (C). For these experiments, we made a few slices from the same tumor block, and imaged the tumor cells using one slice for our “click‐to‐sense” and another for H&E methods. They look slightly different by the different sections, that is the difference caused by the “thickness” of the sections (each consecutively taken from the tumor block by 6 µm), but they are clinically regarded as the same section staining. We defined ROI for the analysis. F) Statistical analysis of cancer cell sensitivity and selectivity among IDC, DCIS, DH, and NBG. Statistical test: *P*‐values, Mann‐Whitney U‐test; Bars, median.

Noteworthy is that by magnifying the fluorescence‐labeled IDC and DCIS images by 20 times, the morphology of IDC and DCIS could be clearly imaged. In order to allow even clearer imaging of the cancer morphology, we obtained images of live tissues from breast cancer patients stained by 20 × 10^−6^ m of “click‐to‐sense” probe **1** in high‐power field (200×). The pathologists diagnosed and compared the double fluorescence staining images (“click‐to‐sense” probe **1** and Hoechst) with the hematoxylin and eosin (H&E) staining images of the same tissue samples in an anonymized form. Images are shown in Figure 4C (IDC, DCIS, DH, and NBG) and Figures S5–S8 (Supporting Information). By staining the breast cancer tissues at a higher fluorescence level than NBG, TAMRA fluorescence derived from our “click‐to‐sense” probe **1** (red) and Hoechst staining images (blue) clearly showed the morphological features of the tissues and the boundaries between IDC, DCIS, DH, and NBG. It should be noted that the fluorescent images of cancers are in good agreement with those of H&E staining (Figure 4C and Figures S5–S8, Supporting Information). Thus, this marks the first successful imaging of cancer morphology at the live tissue level outside of conventional pathology methods.

In order to compare the accuracy of our “click‐to‐sense” chemistry with conventional pathology (H&E stain of permanents sections), we analyzed 30 live cancer tissues (20 IDC and 10 DCIS), 30 adjacent live NBG tissues, and 5 adjacent live DH tissues from 30 breast cancer patients. Following the experiments in Figure 4C, the fluorescence images of all live tissues were expanded (by 20× and by 200×) to image the pathological morphology of cancers. At that magnification, the “click‐to‐sense” chemistry very clearly discriminated IDC, DCIS, DH, and NBG, with the data showing excellent agreement with H&E stain of permanents sections as the conventional pathological method (Figure 4D). It must be noted that diagnosis and discrimination of live cancer tissues only by analyzing the fluorescence intensity would be difficult for DH because a significant proportion of DH fluorescence (mean of 76 at 20 × 10^−6^ m of probe **1**, Figure 4B) overlapped with NBG (mean of 60). However, based on the morphological analysis, it becomes possible to correctly diagnose 3 out of 5 (60%) DH, and 4 out of 5 (80%) non‐malignant lesions (Figure 4D). The diagnosis could be performed more quickly than the conventional method using the time‐consuming frozen section analysis.

To confirm that the fluorescent staining was based on the labeling at the cancer cell level, fluorescent analysis of the frozen sections (6 µm thickness) was further performed with a Zeiss LSM710 confocal microscope in a high‐power field (400×), and then the same frozen sections were also stained with H&E (Figure 4E). We found that breast cancer cells (IDC or DCIS) were fluorescently stained at much higher levels than the cells from NBG tissues in a statistically significant dose‐dependent manner (Figure 4F). In good agreement with the tissue analysis in Figure 4B, the mean fluorescence intensity of DH cells was 72 (Figure 4F), which is between those of DCIS and NBG cells. This value is at the same level as intraductal papilloma, a benign breast condition, (value of 79 in Figure 4E) when they were stained by 20 × 10^−6^ m of probe **1** (Figure 4E). Nevertheless, morphological analysis (Figure S9, Supporting Information) could discriminate intraductal papilloma from DCIS, highlighting the advantage of our “click‐to‐sense” chemistry in terms of its ability to visualize morphology of the lesion.

Likewise, our method could also distinguish the morphology of invasive lobular carcinoma (ILC) (Figure S10, Supporting Information). Thus, various residual carcinomas can be accurately identified using the “click‐to‐sense” probe **1**. This finding shows that live tissue morphology in breast cancer patients can be easily identified, providing support for BCS.

In this paper we showed, using both in vitro and in vivo studies, that our “click‐to‐sense” probe can rapidly discriminate between cancer cells and normal cells, requiring only the staining of live tissues for 5 min, and that it can clearly visualize cancer morphology and localization in a way almost equivalent to frozen section. Our method was not affected by sample conditions, fluorescence backgrounds, or fluorescent spreading, and is not dependent on the time of enzymatic reactions or enzyme expression by cancer cells, as it specifically focuses on the overexpression of endogenously generated acrolein in various cancer cells, with the in‐cell “click‐to‐sense” chemistry successfully anchoring to the fluorescence label in cancer cell constituents. One may think that the immunofluorescence staining could be ideal. But in this research, we should stain the various live “heterogeneous” tumors (and all of them) from breast cancer patients, but not those from the cell and the animal models, where the specific tumor‐associated antigens are expressed. The previous trials of us and the others failed in immunostaining the clinical tumor samples for this reason. Hence our new “click‐to‐sense” method is extremely significant and important, and should be highlighted.

The experiments described in this paper indicate that our method could be used as a new method for margin management of live tissues. The potential utility of our method requires prospective clinical trials for real‐time intraoperative assessment using resection stumps from breast cancer patients. The clinical significance of our probe for morphological and pathological features deserves further study in hospitals worldwide. We speculate that our “click‐to‐sense” chemistry could also be useful for real‐time margin management in gastric cancer, colon cancer, lung cancer, and other cancers in live tissues during surgery, as well as for real‐time margin management of endoscopic polypectomy. It would also be useful to evaluate our method in the diagnosis of axillary lymph node metastasis of breast cancer during surgery. Another potential use of the “click‐to‐sense” chemistry could be to identify circulating tumor cells (CTC).

It would also be useful to consider the use of artificial intelligence (AI) alongside our method. AI has shown potential usefulness as a diagnostic tool in the clinic. In a recent study, AI outperformed pathologists in diagnosing small amounts of cancer that had spread to the lymph nodes in women with breast cancer.^20^ Since our “click‐to‐sense” probe **1** could be used to diagnose cancer tissues during BCS more rapidly than conventional H&E methods, we envision that AI could be used to analyze live tissue stained by probe **1**, increasing the efficiency of real‐time intraoperative assessment.

In conclusion, we were able to show that our “click‐to‐sense” probe **1** clearly and rapidly discriminated breast cancers from NBG in live tissues from patients, not only imaging the presence of cancer lesions but also showing their morphological features. Our new method has the potential to become a new margin management method of live tissues with high selectivity, and could be used as a discriminative, low‐cost, and easy‐to‐perform method for cancer sensing during surgery. The method is going to be confirmed in a clinical prospective study including intraoperative assessment of resection stumps in breast cancer patients.

## Experimental Section

*Detection of Acrolein in Cancer and Normal Cells*: Eleven cell lines (TIG3, HUVEC, MCF10A, SKBR3, MDA‐MB‐231, BxPC3, HT29, MCF7, A549, HeLa S3, and PC3) were seeded on 96‐well plate (2 × 10^4^ cells per well) and left to attach for 24 hours at 37 °C. The cells were then treated with 100 µL of various concentrations of probe **1** solution (2.5 × 10^−6^, 7.5 × 10^−6^, 12.5 × 10^−6^, 17.5 × 10^−6^, 22.5 × 10^−6^ m) in the culture medium, and incubated for 30 min at room temperature. After this incubation, cells were rinsed twice with phosphate‐buffered saline (PBS) and resuspended in 100 µL PBS. The fluorescence intensity was then measured using a spectrofluorometer (SpectraMax M2e, Molecular Devices). Fluorescence intensity was normalized for each cell line by the number of 10 000 cells. The cells were then fixed using 4% paraformaldehyde in PBS for 10 min at room temperature, rinsed with PBS two times, and resuspended in 100 µL PBS. The images of fixed cells were recorded using inverted fluorescence microscope (IX71, Olympus).

*Cancer and Normal Breast Gland Tissues from Breast Cancer Patients*: All tissue samples were taken from surgical resection and informed consent was obtained from each patient. The subjects recruited for this study comprised 30 primary breast cancer patients (20 IDC patients and 10 DCIS patients; mean age: 49.4 years; range: 36–‐67 years) who underwent breast surgery during the period from March 2017 to March 2018 at Osaka University Hospital, Osaka, Japan. This study also involves DH of five patients and intraductal papilloma of two patients.

*Fluorescence Microscopy Analysis of “Click‐to‐Sense” Probe **1** and Hoechst Staining*: The live tissues were cut by Tissue Matrix Chamber, dipped into a 5 × 10^−6^, 10 × 10^−6^, or 20 × 10^−6^ m solution of the “click‐to‐sense” probe **1** in 15 mL conical tubes for 5 min at room temperature, and then rinsed with PBS three times. The nuclei of the resulting tissues were stained with Hoechst 33 342 + 33 258 (Dojindo, Kumamoto, Japan). The tissue samples were then transferred into a glass bottom dish (Thermo Scientific Nunc) and placed in the fluorescence microscope (Keyence BZ‐X710, Osaka, Japan) equipped with optical sectioning algorithm system (to obtain clear images without fluorescence blurring). The fluorescence images of the whole tissue were taken in low‐power field. To evaluate the mean fluorescence intensity of probe **1** (stained red), a regions of interest (ROI) was set in the center of representative lesions (Figure S3, Supporting Information). The average fluorescence intensity of each ROI was calculated and analyzed with Image J software (National Institute of Health, Rockville, MD, USA). The tissue samples were placed in the same excitation light and exposure time for the analysis of both probe **1** (TAMRA) and Hoechst. The fluorescence images of live tissues stained by 20 × 10^−6^ m of probe **1** were taken in high‐power field (200×) for the morphological observation of the tissues samples.

The same live tissues were then embedded in optimal cutting temperature (OCT) compound, freeze, and cut for analysis with confocal microscope (Zeiss LSM710, Göttingen, Germany) in high‐power field (400×). Images were taken from at least three different and randomly chosen views of each sample. To evaluate the mean fluorescence intensity of probe **1** (stained red), ROI was set in the center of representative lesions (Figure 4E). The average fluorescence intensity of each ROI was calculated and analyzed with Image J software. The same tissues were then fixed in 4% paraformaldehyde for immunohistochemical staining. The pathological evaluation was performed by light microscopy analysis of H&E stained sections.

*Statistical Analysis*: The statistical program R (http://cran.r‐project.org) was used for all statistical analysis. Association of mean fluorescence intensity of probe **1** in surgical tissues was evaluated with the Mann‐Whitney U‐test. The statistical significance was assumed for *P* < 0.05.

## Conflict of Interest

The authors declare no conflict of interest.

## Supporting information

Supplementary
