# Supplementary material for: Cascade Reaction in Human Live Tissue Allows Clinically Applicable Diagnosis of Breast Cancer Morphology
Source: Adv Sci (Weinh). 2018 Nov 27;6(2):1801479. doi: 10.1002/advs.201801479 (PMC6343070; doi:10.1002/advs.201801479)
Supplement: Supplementary file 1 — Supplementary [file ADVS-6-1801479-s001.pdf]

Copyright WILEY-VCH Verlag GmbH & Co. KGaA, 69469 Weinheim, Germany, 2018.

Supporting Information

for *Adv. Sci.,* DOI: 10.1002/advs.201801479

Cascade Reaction in Human Live Tissue Allows Clinically Applicable Diagnosis of Breast Cancer Morphology

Tomonori Tanei, Ambara R. Pradipta, Koji Morimoto, Motoko Fujii, Mayumi Arata, Akihiro Ito, Minoru Yoshida, Elena Saigitbatalova, Almira Kurbangalieva, Jun-ichiro Ikeda, Eiichi Morii, Shinzaburo Noguchi, and Katsunori Tanaka**

Copyright WILEY-VCH Verlag GmbH & Co. KGaA, 69469 Weinheim, Germany, 2016.

Supporting Information

Cascade Reaction in Human Live Tissue Allows Clinically Applicable Diagnosis of Breast Cancer Morphology

Tomonori Tanei[†], *Ambara R. Pradipta*[†], *Koji Morimoto, Motoko Fujii, Mayumi Arata, Akihiro Ito, Minoru Yoshida, Elena Saigitbatalova, Almira Kurbangalieva, Jun-ichiro Ikeda, Eiichi Morii, Shinzaburo Noguchi**, *Katsunori Tanaka**

Methods

Total fluorescence intensity of HeLa S3 cell lysates: Hela S3 cells in 15-cm dishes were incubated with probe **1** (10 µM) or control probe without azide group (10 µM, see chemical structure in Figure S2) for 60 minutes at 37 °C. The cells were then rinsed twice with PBS, resuspended in 3 mL PBS, and removed from the dish with a cell scraper. The PBS containing detached cells were transferred into a 15 mL conical tube. The cells were collected by centrifugation at 1,000 rpm for 5 minutes at 4 °C, and the supernatant was removed. The cells were then resuspended in 200 µL RIPA-Buffer, transferred into 1.5 mL microcentrifuge tube, and sonicated on ice for 5 seconds. The mixture was then centrifuge at 1,000 rpm for 5 minutes at 4 °C. The supernatant was transferred into an Amicon 3K centrifuge filter unit and then centrifuge at 15,000 g for 5 minutes at 4 °C. The bottom layer was discarded, and the top layer was diluted with 200 µL of PBS and centrifuge at 15,000 g for 5 minutes at 4 °C. The wash procedure for this top layer was repeated a total of 5 times, and then 10 µL of the remaining residue at the top filter part was transferred into 96-well microplate reader and diluted with 90 µL of H_2O. The fluorescence intensity was measured with spectrofluorometer (FP-6500, JASCO) (Figure 2D-i).

SDS-PAGE: Hela S3 cells were seeded on 6-well plate (1.25×10^6 cells/well) and left to attach for 24 hours at 37 °C. The cells were then treated with probe **1** (1 µM and 10 µM) or control probe without azide group (1 µM and 10 µM) for 60 minutes at 37 °C. After this incubation, cells were rinsed with cold PBS. Whole cell lysates were prepared by collecting cells directly in sodium dodecyl sulfate-polyacrylamide gel electrophoresis (SDS-PAGE) sample loading buffer and heating at 100 °C for 10 minutes. The protein extracts were run on 10% SDS-PAGE, and the luminescence was recorded using biomolecular imager (ImageQuant LAS-4000, GE Healthcare) (Figures 2D-ii and S2-i). The same gel was then stained with coomassie brilliant blue (CBB), and images was obtained by using the digitizing method of the LAS-4000 (Figures 2D-iii and S2-ii).

ROS analysis: The selected cell lines were seeded on 96-well plate (2×10^4 cells/well) and left to attach for 24 hours at 37 °C. ROS production was measured by the total ROS detection kit (ROS-ID®, Enzo) (Figure 2E-i).

FDP lysine analysis: The cells grown in 10 cm dishes were washed with PBS and resuspended in 1 mL PBS. Cells at confluence were detached by treatment with 0.1 mL of trypsin/EDTA solution. The PBS containing detached cells were transferred into a 1.5 mL microcentrifuge tube. The cells were collected by centrifugation at 1,000 rpm for 5 min at 4 °C. After the supernatant was removed, the cells were resuspended in 100 µL RIPA-Buffer, and sonicated on ice for 15 seconds. About 20 µL of the RIPA-Buffer containing cells were transferred into 1.5 mL microcentrifuge tube and diluted with 380 µL of H_2O. In a new and clean 1.5 mL microcentrifuge tube, 5 µL of the diluted cells, 25 µL of DMF/H_2O (1:1), and 25 µL of nitroarene probe*) was mixed and heated to 100 °C for 5 hours. The crude mixture was then diluted with 50 µL H_2O, and transferred into 96-well microplate reader. The fluorescence intensity was measured with spectrofluorometer (FP-6500, JASCO) at 340 nm / 404 nm (Figure 2E-ii).

*) Note: Nitroarene probe was prepared by mixing 4-nitrobenzoic acid (340 mg) and $CaCl_2$ (1.10 mg) in 10 mL of DMF/H_2O (1:1).[18]

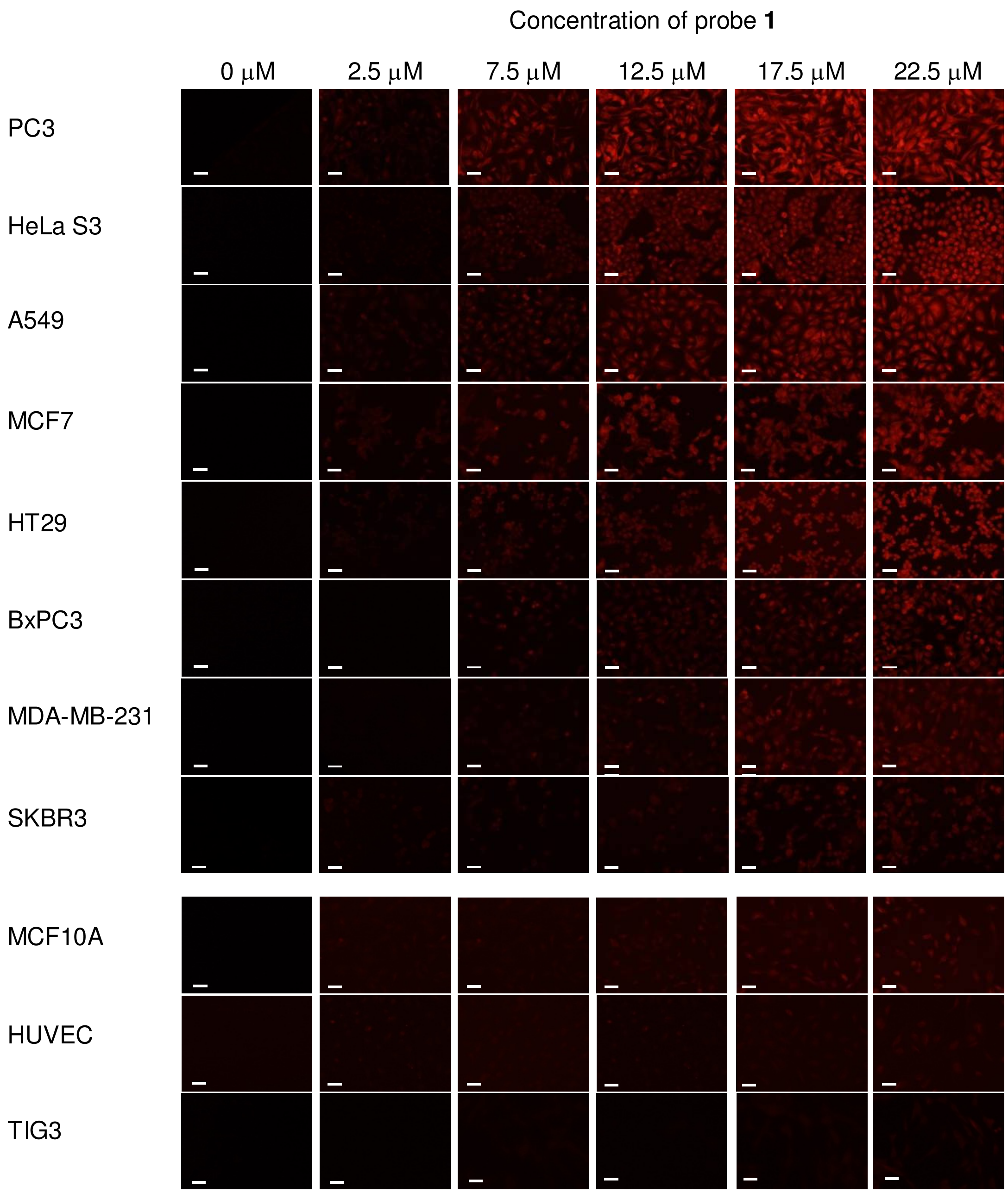

Figure S1. Discrimination of cancer cells (PC3, HeLa S3, A549, MCF7, HT29, BxPC3, MDA-MB-231, SKBR3) from normal cells (MCF10A, HUVEC, TIG3) by "click-to-sense" probe **1**. Eleven cell lines were treated with various concentration (0, 2.5, 7.5, 12.5, 17.5, and 22.5 μM) of probe **1** at 37 °C for 30 minutes. The cells were fixed and fluorescence was recorded by SpectraMax M2e, Molecular Devices. The scale bar indicates 10 μm.

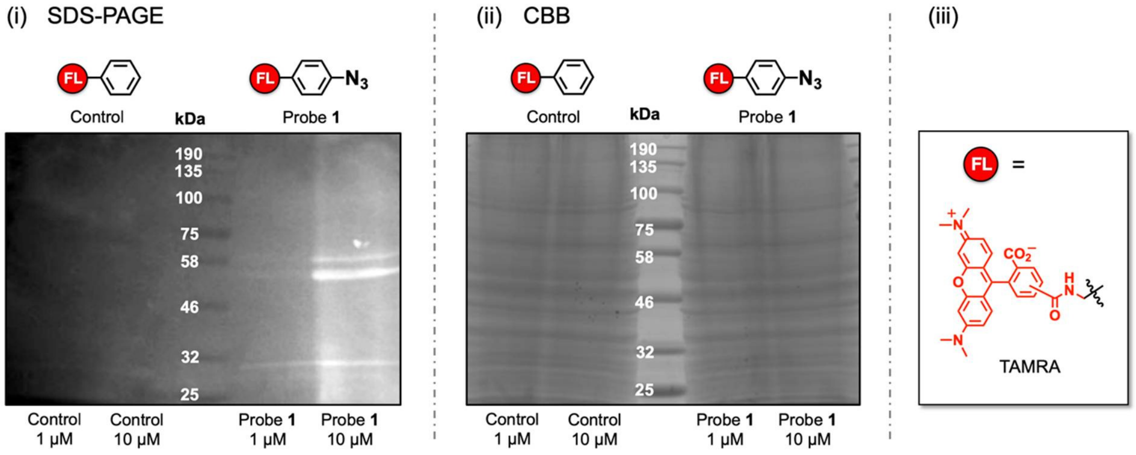

Figure S2. (i) The SDS-PAGE images by fluorescence detection of HeLa S3 cell lysates treated with probe **1** (1 μM and 10 μM) and control probe without azide group (1 μM and 10 μM). (ii) The SDS-PAGE by coomassie staining. (iii) The chemical structure of TAMRA fluorescence.

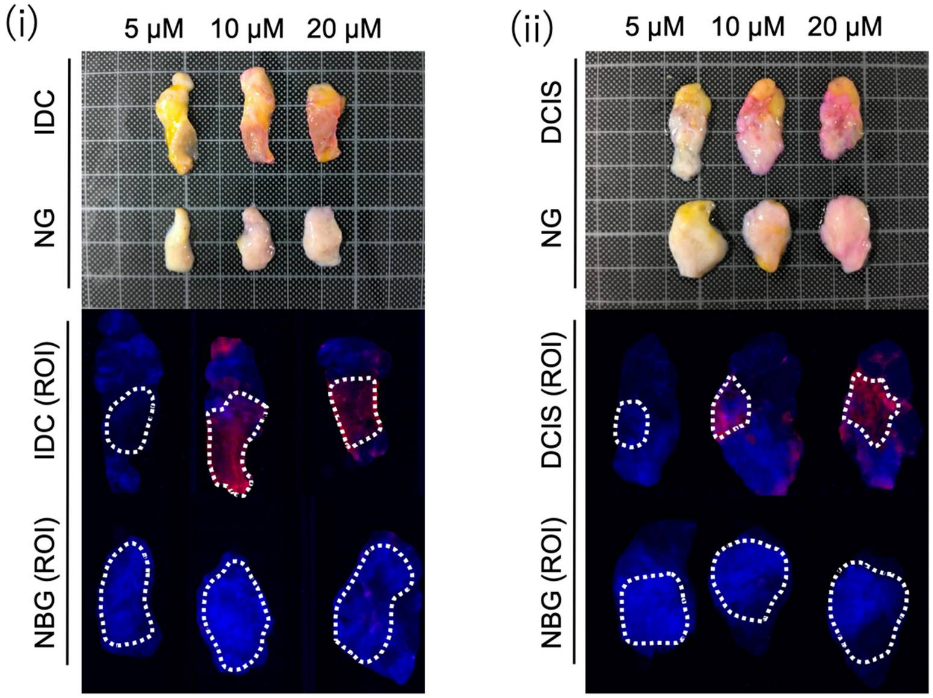

Figure S3. Representative pictures and double-staining microscopic images of (i) IDC and NBG, (ii) DCIS and NBG. To evaluate the mean fluorescence intensity of probe **1** (stained red), ROI was set in the center of representative lesions. The average fluorescence intensity of each ROI was calculated and analyzed with Image J software.

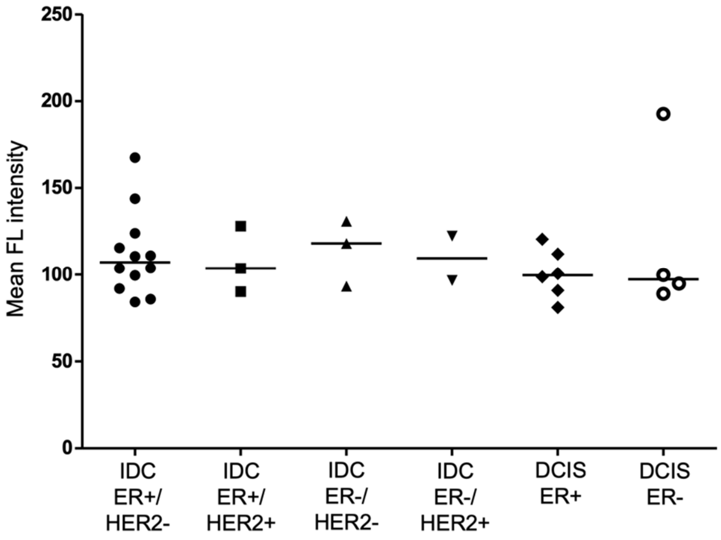

Figure S4. Statistical analysis of cancer sensitivity and selectivity among IDC (ER+/HER2-; ER+/HER2+; ER-/HER2-; ER-/HER2+) and DCIS (ER+; ER-).

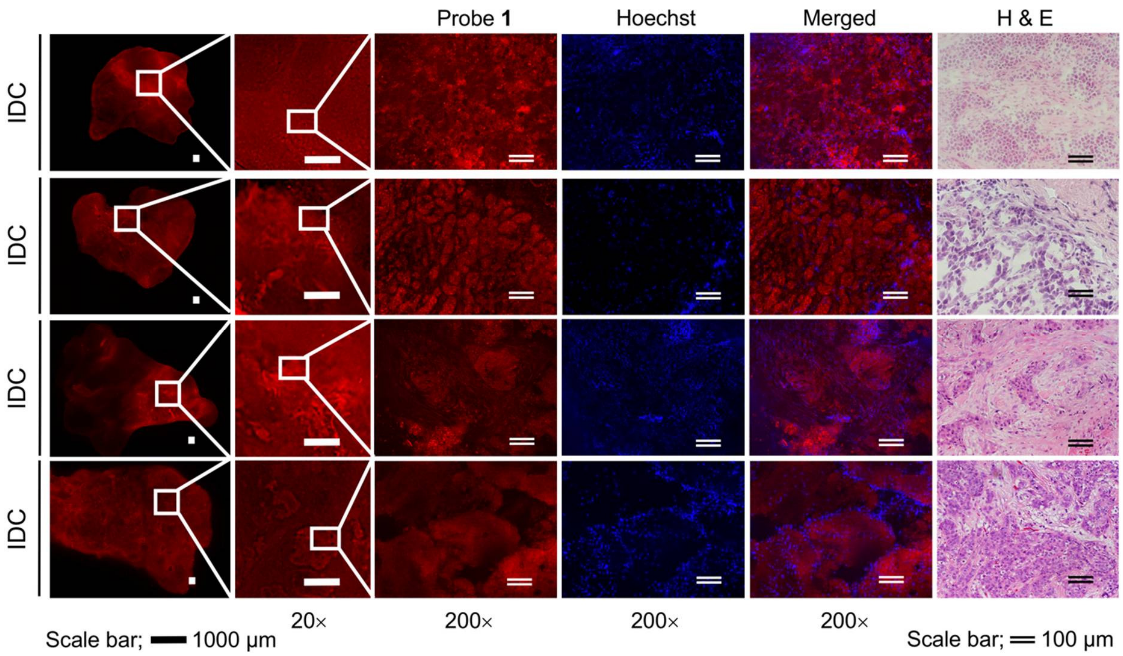

Figure S5. Expansion of images; Morphology of IDC can clearly be detected by 20× and 200× (highly magnified fluorescent images) being labeled by probe **1** at 20 μM. The detection of cancer morphology was consistent with H&E staining images of the frozen sections prepared from the same anonymized tissue samples.

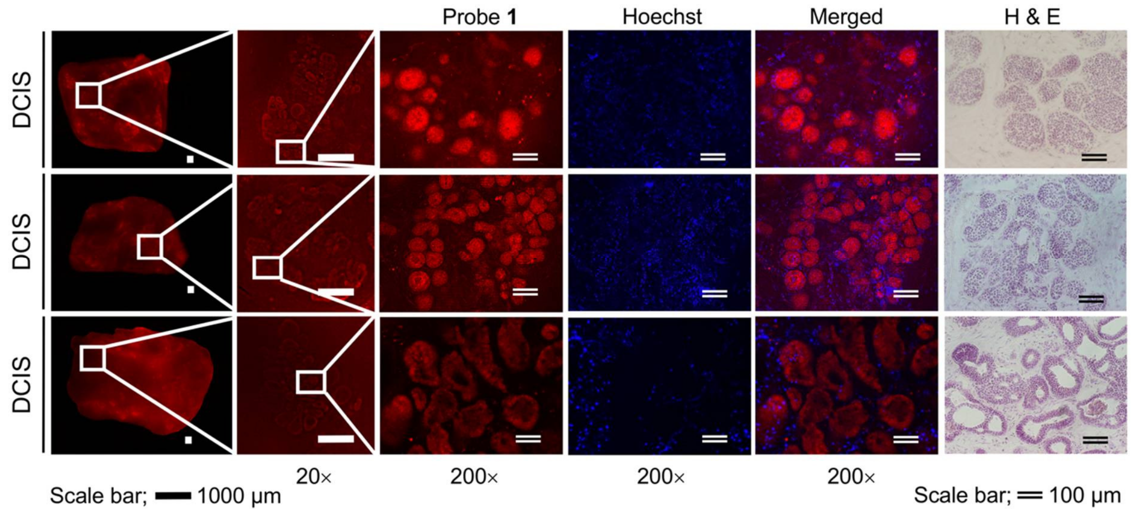

Figure S6. Expansion of images; Morphology of DCIS can clearly be detected by 20× and 200× (highly magnified fluorescent images) being labeled by probe **1** at 20 μM. The detection of cancer morphology was consistent with H&E staining images of the frozen sections prepared from the same anonymized tissue samples.

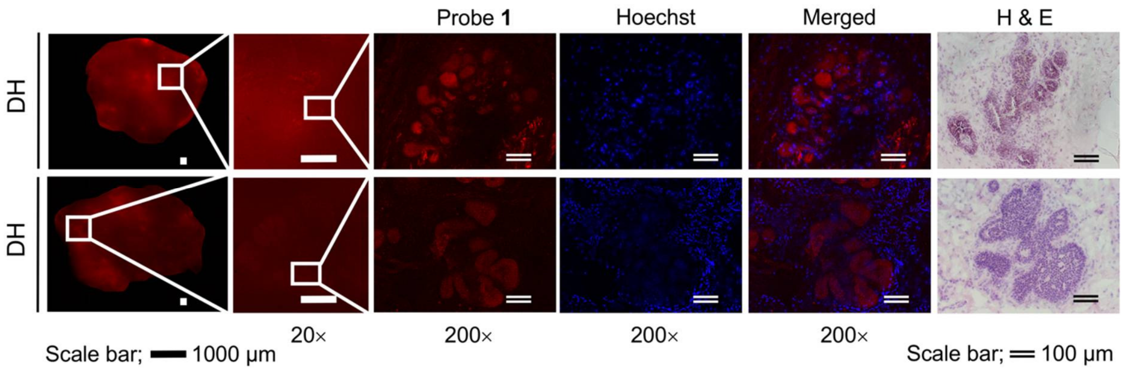

Figure S7. Expansion of images; Morphology of DH can clearly be detected by 20× and 200× (highly magnified fluorescent images) being labeled by probe **1** at 20 μM. The detection of cancer morphology was consistent with H&E staining images of the frozen sections prepared from the same anonymized tissue samples.

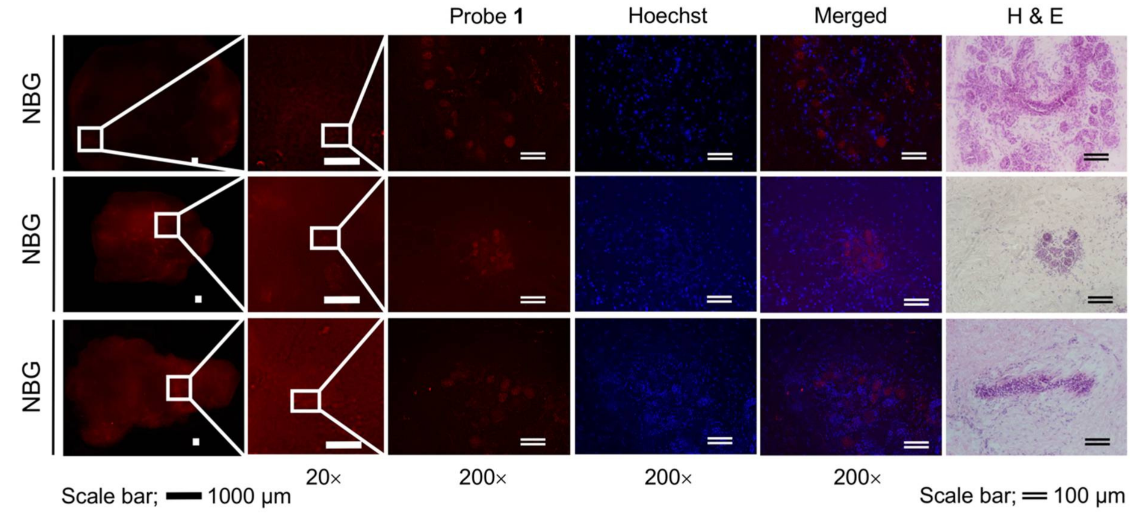

Figure S8. Expansion of images; Morphology of NBG can clearly be detected by 20× and 200× (highly magnified fluorescent images) being labeled by probe **1** at 20 μM. The detection of cancer morphology was consistent with H&E staining images of the frozen sections prepared from the same anonymized tissue samples.

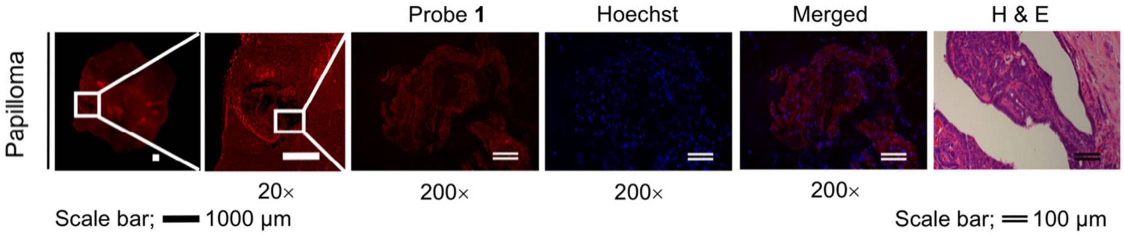

Figure S9. Microscopic images (20× and 200×) from the sample tissues labeled by probe **1** at 20 μM. The "click-to-sense" method can also be used to detect morphology and distinguish breast intraductal papilloma (a benign breast condition). Confocal microscopic images (400×) of the frozen sections from breast intraductal papilloma are shown in Figure 4E. The detection of cancer morphology was consistent with H&E staining images of the frozen sections prepared from the same anonymized tissue samples.

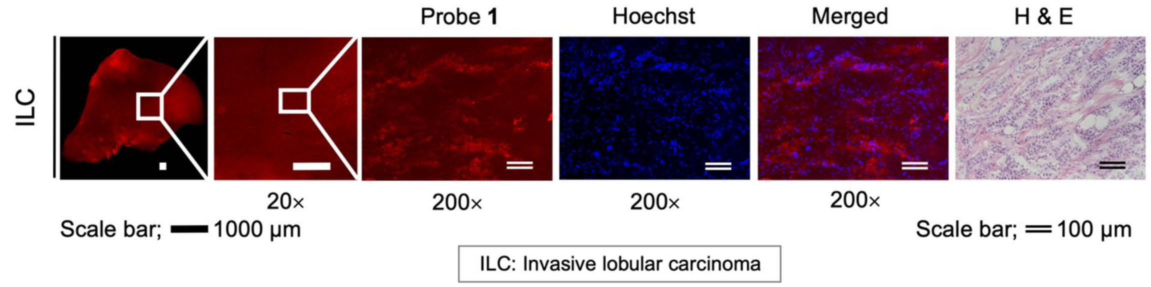

Figure S10. Microscopic images (20× and 200×) from the sample tissues labeled by probe **1** at 20 µM. The "click-to-sense" method can also be used to detect morphology and distinguish invasive lobular carcinoma (ILC, the second most common type of breast cancer after IDC). The detection of cancer morphology was consistent with H&E staining images of the frozen sections prepared from the same anonymized tissue samples.

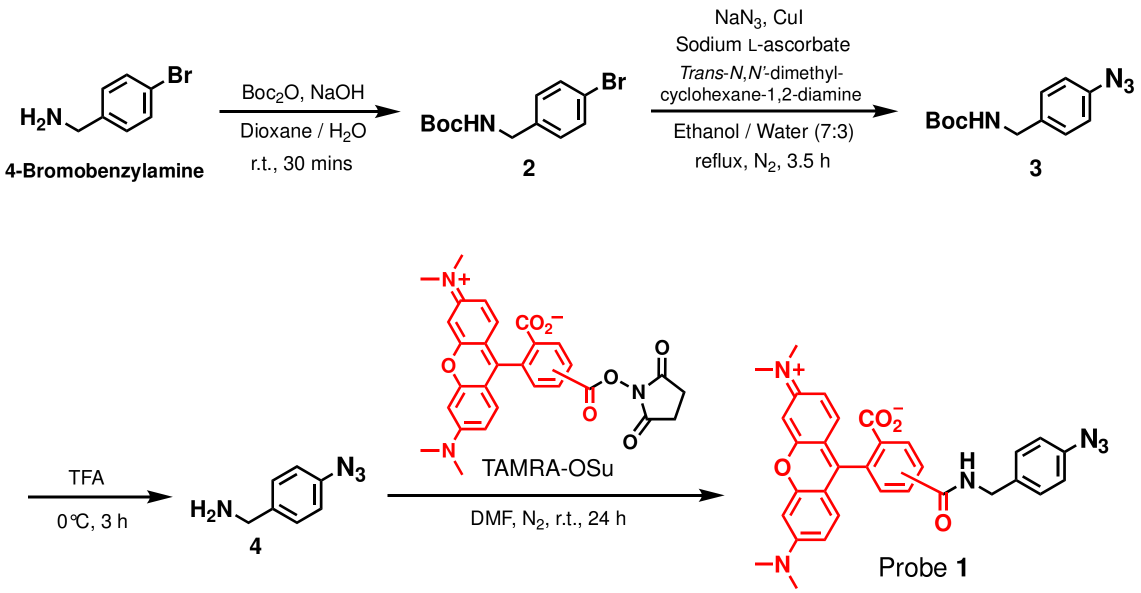

Figure S11. The synthesis scheme of probe **1**.

*Procedure for the synthesis of fluorescence labeled phenyl azide (probe **1**)*[15]

The 4-bromobenzylamine (748 µL, 1.10 g, 5.90 mmol), Boc_2O (1.9 g, 8.9 mmol), and NaOH (355 mg, 8.90 mmol) were stirred in dioxane/water (1.5:1) (25 mL, [4-bromobenzylamine] = 0.2 M) at ambient temperature. After being stirred for 30 minutes, the solvents were removed by rotary evaporation. The residual crude was taken up with chloroform, and washed with saturated NH_4Cl solution. The organic layers were dried over Na_2SO_4, filtered and concentrated to dryness under reduced pressure to give compound **2** as colorless crystal (1.4 g, 82%).

Compound **2** obtained above (227 mg, 790 µmol), NaN_3 (155 mg, 2.38 mmol), sodium L-ascorbate (8.0 mg, 40 µmol), CuI (15 mg, 80 µmol), and *trans*-*N*,*N'*-dimethylcyclohexane-1,2-diamine (26.0 µL, 23.0 mg, 160 µmol) were stirred in ethanol/water (7:3) (8.0 mL, [**2**] = 0.1 M). The reaction mixture was capped under N_2 atmosphere and refluxed for 3.5 h. After removal of ethanol by rotary evaporation, the mixture was extracted with chloroform. The organic layers were combined, washed with brine, dried over Na_2SO_4, filtered and concentrated to dryness under reduced pressure. The crude product was purified by silica gel chromatography using hexane–EtOAc (4:1) as the eluent to give the product compound **3** as pale-yellow crystal (160 mg, 81%).

Compound **3** obtained above (16 mg, 65 µmol) in TFA (600 µL, [**3**] = 0.1 M) was stirred vigorously at 0°C. After being stirred for 3 h, the pH was adjusted to 8 by adding 10 M NaOH solution and then extracted with $CHCl_3$. The organic layers were combined, washed with brine, dried over Na_2SO_4, filtered and concentrated to dryness under reduced pressure to give the crude product compound **4** as dark yellow oil (8.3 mg, 87%).

Compound **4** prepared above (5.3 mg, 36 µmol) and TAMRA-OSu (18.9 mg, 35.8 µmol) were stirred in anhydrous DMF (720 µL, [**4**] = 0.05 M) under N_2 atmosphere at ambient temperature. After being stirred for 24 h, DMF was removed azeotropically with toluene by rotary evaporation. The crude product was purified by reversed-phase HPLC to give the product fluorescence labeled phenyl azides (probe **1**) as dark purple solid (12 mg, 60%).

Conditions of reversed-phase HPLC: Column, Cosmosil 5C$_{18}$-AR300 (Nacalai Tesque, Inc.) 10×250 mm; Mobile phase A, 0.1% TFA in H_2O; B, 0.1% TFA in CH_3CN; Gradient elution, 0-4 min at 50% B, 4-14 min at 50-80% B, 14-15 min at 80% B; Flow rate at 4 mL/min; UV detection at 254 nm.
Probe **1**, dark purple solid. IR (neat, cm^{-1}): ν 3420, 2942, 2108, 1595, 1348, 1188, 1136. ^{1}H NMR (400 MHz, CD_3CN, 25 °C): δ 8.70 (s, 1H), 8.35-8.11 (m, 1H), 7.96-7.76 (m, 1H), 7.43 (d, J = 8.2 Hz, 1H), 7.37 (d, J = 8.4 Hz, 1H), 7.12-6.92 (m, 7H), 6.84 (d, J = 1.8 Hz, 2H), 4.55 (dd, J = 30.4, 5.4 Hz, 2H), 3.25 (s, 12H). ESI-HRMS *m/z* calcd for $C_{32}H_{29}N_6O_4$ ($[M+H]^+$) 561.2245, found 561.2243.
